# Recent progress on harm, pathogen classification, control and pathogenic molecular mechanism of anthracnose of oil-tea

**DOI:** 10.3389/fmicb.2022.918339

**Published:** 2022-07-29

**Authors:** Xinggang Chen, Xingzhou Chen, Qian Tan, Xiuli Mo, Junang Liu, Guoying Zhou

**Affiliations:** Key Laboratory of National Forestry and Grassland Administration for Control of Diseases and Pests of South Plantation, Hunan Provincial Key Laboratory for Control of Forest Diseases and Pests, Key Laboratory for Non-wood Forest Cultivation and Conservation of Ministry of Education, Central South University of Forestry and Technology, Changsha, China

**Keywords:** anthracnose, oil tea, *Colletotrichum* spp., *Camellia oleifera*, pathogenic molecular mechanism

## Abstract

Oil tea (*Camellia oleifera*), mainly used to produce high-quality edible oil, is an important cash crop in China. Anthracnose of oil tea is a considerable factor that limits the yield of tea oil. In order to effectively control the anthracnose of oil tea, researchers have worked hard for many years, and great progress has been made in the research of oil tea anthracnose. For instance, researchers isolated a variety of *Colletotrichum* spp. from oil tea and found that *Colletotrichum fructicola* was the most popular pathogen in oil tea. At the same time, a variety of control methods have been explored, such as cultivating resistant varieties, pesticides, and biological control, etc. Furthermore, the research on the molecular pathogenesis of *Colletotrichum* spp. has also made good progress, such as the elaboration of the transcription factors and effector functions of *Colletotrichum* spp. The authors summarized the research status of the harm, pathogen types, control, and pathogenic molecular mechanism of oil tea anthracnose in order to provide theoretical support and new technical means for the green prevention and control of oil tea anthracnose.

## Introduction

### Cultivation and application of oil tea

Oil tea generally refers to the *Camellia* genus, which has rich seed oil content that is produced and highly valuable (Chen, 2008). The history of extracting oil from oil teaseed in China can be traced back to 2,300 years ago (Zhuang, 2008). The main varieties of oil tea cultivated in China include *Camellia yuhsienensis* Hu, *Camellia oleifera* var *monosperma, Camellia vietnamensis*, and *Camellia oleifera*, among which the planting range of *Ca. oleifera* is the widest (Qin et al., 2018; Chen et al., 2021b). Tea oil extracted from the seed of oil tea is rich in unsaturated fatty acids and vitamin E and has unique nutritional value (Shi et al., 2020). Thus, oil tea is as famous as coconut, palm, and olive, and is also known as one of the four major woody oil plants in the world (Yang et al., 2016, 2020a). Moreover, the United Nations Food and Agriculture Organization (FAO) recommended tea oil as a high-quality and healthy vegetable oil, owing to its nutritional value and excellent storage quality (Chen et al., 2020b). In 2020, the area in China planted oil tea reached 45,333.3 km^2^; the output of tea oil reached 627,000 tons, and the output value of the tea oil industry reached 18 billion U.S. dollars, indicating that tea oil is highly valuable (Chen et al., 2021a).

### Anthracnose of oil tea

Anthracnose of oil tea is a considerable factor that limits the yield of tea oil (Chen et al., 2020a; Li et al., 2021b). Anthracnose of oil tea is a considerable factor that limits the yield of tea oil (Chen et al., 2020a; Li et al., 2021b). The conidia of *Colletotrichum* spp. mainly infect the oil tea from the wound but also through the natural pore, such as stoma. Attached conidia germinate and differentiate dome-shaped appressoria on plant surfaces, underneath which penetration pegs form and penetrate epidermal cells. The pathogen then differentiates bulbous biotrophic hyphae, which are enveloped by an intact host plasma membrane; biotrophic hyphae spread across living host cells before differentiating thin necrotrophichyphae that kill and destroy host tissues (Münch et al., 2008; O'Connell et al., 2012).

*Colletotrichum* spp. primarily infect the leaves and fruits of the oil tea, leading to a 20–40% fruit drop and up to 40% seed loss (Jin et al., 2009; Zhu et al., 2015). In addition, the oil content of oil tea seeds infected by *Colletotrichum* spp. can be reduced by 50%. Moreover, the anthracnose of oil tea can also cause the germination of infected seeds, which facilitates the long-distance transmission of oil tea anthracnose (Yang, 2009). Therefore, under the conditions of appropriate humidity and ambient temperature, anthracnose of oil tea spreads rapidly and is difficult to control, causing substantial economic losses and seriously damaging the safety of edible oil in China (Liu et al., 2009; Deng, 2011). *Colletotrichum* spp. are also regarded as among the top 10 plant pathogenic fungi in the field of molecular plant pathology because of their strong pathogenicity and wide spread (Dean et al., 2012).

*Colletotrichum* spp. was first discovered by Tode in 1790 and named *Vermicularia* Tode (Tode, 1790). Then, it was further subdivided according to other morphological characteristics and named *Colletotrichum* Corda (Sturm, 1832; Sutton, 1992). With the development of molecular biology, more and more scholars used multi-gene lineage to identify *Colletotrichum* spp., which not only improved the accuracy of *Colletotrichum* spp. identification but also identified more species of *Colletotrichum* spp. Talkin has identified *Colletotrichum* spp. according to internal transcribed spacer (ITS), histone 4 (HIS4), and β-tubulin 2 (TUB2) gene polygenic sequences for the first time (Talhinhas et al., 2002).

Nowadays, more and more *Colletotrichum* spp. has been identified, and the *Colletotrichum* spp. that can infect oil tea mainly include *Colletotrichum fructicola, Colletotrichum gloeosporioides, Colletotrichum horii, Colletotrichum siamense, Colletotrichum camelliae*, and *Colletotrichum boninense* (Li et al., 2014, 2017). Approximately, 406 strains of *Colletotrichum* spp. were isolated from oil tea in 10 provinces of China by Li. The results showed that *Co. fructicola* was the most widely distributed in oil tea, so the prevention and the control of *Co. fructicola* were one of the key points of oil tea anthracnose control (Li, 2018). *Co. fructicola* is widely distributed and has many hosts, such as oil tea, apple, strawberry, mango, banana, coffee, and other plants of more than 50 species, among which oil tea is one of its main hosts (Prihastuti et al., 2009; Weir et al., 2012; Huang et al., 2013; Li et al., 2013; Diao et al., 2017). Although anthracnose of oil tea has attracted more and more attention, there are few effective control methods. The reason is that the pathogenic mechanism of *Colletotrichum* spp. and the immune mechanism of the host are not well understood.

### Control of anthracnose of oil tea

The control of oil tea anthracnose can be divided into prevention and treatment. Breeding and planting resistant plants are important measures to prevent plant disease (Savchenko, 2017). *Ca.yuhsienensis* Hu, a species of oil tea, was once widely cultivated in central China because of its high quality, yield, and high resistance to anthracnose (Denton-Giles et al., 2013; Denton-Giles, 2014; Cao et al., 2017; Nie et al., 2020). In contrast to *Ca. oleifera*, which has the largest planting area, *Ca. yuhsienensis* is not generally infected by *Colletotrichum* spp. Yang et al. (2004) and Duan et al. (2005) found that *Ca. yuhsienensis, Camellia octopetala* Hu, *Ca. oleifera* Abel var. Huizhou-xiaohong and *Ca. oleifera* Abel var. Huizhou-dahong have resistance to *Colletotrichum* spp. (Yang et al., 2004; Duan et al., 2005). Moreover, *Ca. yuhsienensis* also showed strong resistance to other pathogens, such as *Ciborinia camelliae* (Denton-Giles et al., 2013; Denton-Giles, 2014; Saracchi et al., 2019; Li et al., 2020). Consequently, *Ca. yuhsienensis*, as a wild relative of *Ca. oleifera*, is widely used to breed varieties of oil tea (Nie et al., 2020).

After selecting suitable oil tea varieties, the seedling quarantine should be strictly controlled. When selecting seedlings and other reproductive materials, quality inspection must be carried out in accordance with national and regional standards to ensure the safety of various reproductive materials (Shan et al., 2019). After planting oil tea, the cultivation management should be strengthened to create environmental conditions that are not conducive to the survival, reproduction, and transmission of pathogen and are suitable for the growth of oil tea (Shu and Zhang, 2009).

When oil tea was infected with *Colletotrichum* spp., the treatment of anthracnose is primarily based on the use of chemical pesticides. For oil tea forest in the early stage of the anthracnose, Bordeaux mixture can be used for treatment. For oil tea forest in the late stage of the anthracnose, chlorothalonil, carbendazim, and thiophanate methyl can be used for treatment (Yu, 2019).

However, the abuse of pesticides not only easily causes environmental pollution but also leads to the emergence of pathogens resistant to pesticides (Holtappels et al., 2021). Therefore, biological control has been a hot spot in the research of oil tea anthracnose in recent years. *Bacillus subtilis* Y13 was isolated from healthy leaves of *Ca. oleifera* by Zhou. The results showed that its inhibitory rate on *Colletotrichum* spp. was 88.5% (Zhou et al., 2008). *Bacillus velezensis* HBMC–B05 was isolated from healthy leaves of *Ca. oleifera* by Shang. The results showed that its inhibitory rate on *Colletotrichum* spp. was 88.5% (Shang et al., 2021). Yu. (2019) further found that lipopeptides, various metabolites of antagonistic bacteria, had antagonistic effect on *Colletotrichum* spp. (Yu, 2019). Moreover, many strains were isolated, such as *Bacillus subtilis* R6, *Paenibcillus kribbens* Z17, *Brevibacillus brevis* Z26, *Streptomvces globisporus* subsp *Globisporus* F10, and *Streptomyces albulus* cf17. It was found that these strains could inhibit the growth of *Colletotrichum* spp. (Song et al., 2012).

In addition to antagonizing microorganisms, some elicitors can also improve the resistance of oil tea to *Colletotrichum* spp., such as salicylic acid (SA), methyl jasmonate (MeJA), and so on (Wang et al., 2022). Although the research on the control of oil tea anthracnose has made good progress, the anthracnose still puzzles the oil tea industry. The main reason is that the pathogenic mechanism of oil tea anthracnose has not been fully proved, which is difficult to provide theoretical guidance for the prevention and control of oil tea anthracnose. Therefore, to understand the pathogenic mechanism of oil tea anthracnose, the molecular mechanism of the interaction between plants and pathogens needs to be understood.

### Pathogenic mechanism of anthracnose

The plant immune system includes pathogen-associated molecular pattern-triggered immunity (PTI) and effector-triggered immunity (ETI) (Jones and Dangl, 2006). PTI is triggered by plasma membrane-localized pattern recognition receptors (PRRs) specific recognition of pathogen-associated molecular pattern (PAMP) or damage-associated molecular patterns (DAMPs) (Dangl et al., 2013; Lo Presti et al., 2015; Ranf, 2017). Pathogens secrete effectors to inhibit PTI to promote infection. The resistance gene (R gene) of the host that can be activated by effectors induces hypersensitive reaction (HR) or programmed cell death (PCD) to inhibit the growth of pathogens (Jones and Dangl, 2006). PTI and ETI jointly limit the invasion of pathogens, and pathogens secrete new effectors again to promote infection (Ngou et al., 2021; Yuan et al., 2021). This is the “zigzag” plant immune model proposed by Jones, which expounds the molecular mechanism of the interaction between pathogens and plants (Jones and Dangl, 2006).

These inducible defenses are also associated with wide-ranging transcriptional and hormonal reprogramming in plants (Pieterse et al., 2012; Chen and Ding, 2020). For instance, hormones also play an important role in plant immune regulation. Among them, SA and jasmonic acid (JA) are considered as the main defense hormones, while others such as gibberellin (GAs), ethylene (ET), abscisic acid (ABA), brassinosteroids (BRS), auxin [indole-3-acetic acid (IAA)], cytokinin (CK), and nitric oxide (NO) are also the regulators of plant immune signal network (Browse, 2009; Pieterse et al., 2012; Wang et al., 2020b; Yang et al., 2020b; Zheng et al., 2020). These pathways and signals together construct the plant immune system.

*Colletotrichum* spp. is widely distributed and extremely destructive, and can infect almost all plants. Therefore, the pathogenic mechanism of anthracnose has always been a research hotspot (Hyde et al., 2009; Cannon et al., 2012). Great progress has been made in the characterization of a single pathogenic gene (Table 1). As early as 1999, Geffroy et al. (1999) found that the R gene of *Phaseolus vulgaris* has different resistance to *Colletotrichum lindemuthianum* from different sources. This result further expounded the relationship between *Colletotrichum* spp. and plant immunity (Geffroy et al., 1999). Subsequently, new R genes were found one after another. These results deepened the understanding of “gene to gene” theory (Melotto and Kelly, 2001; López et al., 2003; Zou et al., 2018). With the deepening of research, great progress has been made in the exploration of *Colletotrichum* spp., such as the elucidation of protein phosphorylation and dephosphorylation, mitogen-activated protein kinase (MAPK), and calmodulin signal, which is helpful to understand the growth and pathogenesis of *Colletotrichum* spp. (Dickman and Yarden, 1999; Kim et al., 2000; Takano et al., 2000; Chen and Dickman, 2002, 2004; Ha et al., 2003; Liang et al., 2019; Zhang et al., 2019).

**Table 1:** The partial genes currently characterized in *Colletotrichum* spp.

**Pathogen**	**Host**	**Gene name**	**Describes**	**References**
*Co.fructicola*	Oil tea (*Ca. oleifera*)	*STE50*	A scaffold protein that mediates vegetative growth, asexual reproduction, appressorium formation, pathogenicity and the response to external stress	Chen et al., 2020a
*Co.fructicola*	Oil tea (*Ca. oleifera*)	*CfVam7*	A SNARE protein that mediates growth, endoplasmic reticulum stress response, and pathogenicity	Li et al., 2021a
*Co.fructicola*	Oil tea (*Ca. oleifera*)	*CfVps39*	A HOPS protein that mediates appressorium formation, environmental stress response and vacuolar fusion	Li et al., 2021b
*Co.fructicola*	Oil tea (*Ca. oleifera*)	*CfHac1*	A transcription factor that mediates growth and pathogenesis	Yao et al., 2019
*Co.fructicola*	Oil tea (*Ca. oleifera*)	*CfGcn5*	An enzyme that mediates growth, development, and pathogenicity	Zhang et al., 2021
*Co.fructicola*	Oil tea (*Ca. oleifera*)	*CfGcn5*	A key component of the AMPK (AMP-activated protein kinase) pathway	Zhang et al., 2019
*Co.fructicola*	Apple (*Gala variety*)	*CfPMK1*	A MAP kinase that mediates pathogenesis, development, and stress tolerance	Liang et al., 2019
*Co.fructicola*	Apple	*CfSte12*	A transcription factor that mediates early apple glomerella leaf spot pathogenesis	Liu et al., 2020
*Co.fructicola*	Strawberry (*Fragaria* × *ananassa* Duch)	*CfShy1*	An effector interfering with salicylic acid accumulation	He et al., 2019
*Co. gloeosporioides*	*Cunninghamia lanceolata, Populus* × *euramericana*cv. “Nanlin895” and *Liriodendron chinensis* × *tulipifera*	*CgCrzA*	A transcription factor that mediates cell wall integrity and infection-related morphogenesis	Wang et al., 2020a
*Co. gloeosporioides*	Avocado	*CgMEK1*	A MAP kinase that mediates pathogenesis	Kim et al., 2000
*Co. gloeosporioides*	*Stylosanthesguianensis*	*CgDN3*	A pathogenicity protein associated with the biotrophic phase of primary infection and required to avert a hypersensitive-like response by a compatible host	Stephenson et al., 2000
*Co. higginsianum*	*Arabidopsis thaliana*	*ChELP1 and ChELP2*	an effector containing LysM motifs which play dual roles in appressorial function and suppression of chitin-triggered plant immunity	Takahara et al., 2016
*Co.lindemuthianum*	Bean (*Phaseolusvulgaris*)	*CIH1*	An effector containing LysM motifs which may function in chitin sequestration and camouflage	de Jonge and Thomma, 2009
*Co. orbiculare*	Cucumber and *Nicotiana benthamiana*	*CoNIS1*	An effector suppresses PAMP-triggered immunity by targeting plant immune kinases	Yoshino et al., 2012; Irieda et al., 2019
*Co. siamense*	*Rosa chinensis*	*CsBzip10*	A transcription factor that mediates vegetative growth, asexual development, appressorium formation and pathogenicity	Liu and Li, 2019
*Co. scovillei*	*Piper nigrum*	*CsHOX1- CsHOX10*	transcription factors that mediate fungal development and the suppression of host defense	Fu et al., 2021

Transcription factors also play a very important role in the growth and pathogenesis of *Colletotrichum* spp. Wang et al. (2020a) found that calcineurin-resonsive transcription factor *CgCrzA* was not only involved in regulating cell wall integrity but also in morphogenesis and virulence in *Co. gloeosporioides*, which proved the importance of the calmodulin signal to *Colletotrichum* spp. (Wang et al., 2020a). Liu found that the transcription factor *CsBzip10* controls vegetative growth, asexual development, appressorium formation, and pathogenicity in *Co. siamense* (Liu and Li, 2019). Fu also found 10 transcription factors that affect growth and inhibit host immunity from *Colletotrichum scovillei* (Fu et al., 2021). Moreover, many important transcription factors have been found in *Co. fructicola*. Yao found that transcription factor *CfHac1* plays critical roles in growth, conidiation, appressorium formation, and pathogenicity, and respond to osmotic stress in *Co. fructicola* (Yao et al., 2019). Li found that transcription factor *CfVam7* is required for growth, endoplasmic reticulum stress response, and pathogenicity of *Co. fructicola* (Li et al., 2021a). In addition to the transcription factors introduced above, the functions of transcription factors, such as *CfSte12* and *CfSte50* of *Co. fructicola*, have also been explored, which lays a foundation for understanding the pathogenesis of anthracnose (Chen et al., 2020a; Liu et al., 2020).

*Colletotrichum* spp. can also secrete effectors to promote infection. As early as 1994, the effector *CIH1*, an effector containing tandem chitin-binding lysin motifs (LysM), which may function in chitin sequestration and camouflage, was found in *Colletotrichum lindemuthianum* (Pain et al., 1994; Perfect et al., 1998; de Jonge and Thomma, 2009; Stergiopoulos and Wit, 2009). Subsequently, de Queiroz systematically predicted and obtained several effectors of *Co. lindemuthianum* (de Queiroz et al., 2019). Takahara et al. (2009, 2016) also found effectors with LysM from *Colletotrichum higginsianum*; results suggested a dual role for these LysM proteins as effectors for suppressing chitin-triggered immunity and as proteins required for appressorium function (Takahara et al., 2009, 2016). Subsequently, Kleemann et al. (2008, 2012) also identified multiple effectors from *Co. higginsianum*, and the results showed that most effectors are host induced and expressed in consecutive waves associated with pathogenic transitions, indicating distinct effector suites are deployed at each stage (Kleemann et al., 2008, 2012). Yoshino et al. (2012) and Irieda et al. (2019) found that the effector NIS1 of *Colletotrichum orbiculare* can interact with PRRs of plants to inhibit immunity (Yoshino et al., 2012; Irieda et al., 2019). Further studies found that the *CgDN3* gene, which can inhibit the function of NIS1, is an important gene to maintain the pathogenicity of *Co. gloeosporoides* (Stephenson et al., 2000). Eisermann et al. (2019) also found two important effectors from *Colletotrichum graminicola* (Eisermann et al., 2019). Schmidt et al. (2020) Andree found that reactive oxygen species (ROS) can increase the resistance of *Arabidopsis* to *Co. higginsianum*; the results further proved the important role of ROS in plant immunity (Schmidt et al., 2020). On the contrary, the *CfShy1* and *CfGcn5* effectors of *Co. fruticola* will affect the homeostasis of host SA and inhibit plant immunity (He et al., 2019; Zhang et al., 2021).

In recent years, in addition to exploring the function of a single gene of *Colletotrichum* spp., some scholars have also used omics methods to explore *Colletotrichum* spp. RNA-seq has been used to study *Colletotrichum*–host interactions. Previous studies have explored the transcriptional profile of *Colletotrichum* spp. after being infected by the host; the results showed that small secreted proteins (SSPs), cytochrome P450s, carbohydrate-active enzymes (CAZYs), and secondary metabolite (SM) synthetases were enriched (Liang et al., 2018). There are also studies to explore the transcriptional profile of a host after *Colletotrichum* spp. infection revealed that many genes were mainly related to immune response, plant hormone signal transduction, and secondary metabolites (Fang et al., 2021; Mehmood et al., 2021). Some studies have discussed the simultaneous response between *Colletotrichum* spp. and hosts, which provides a new perspective for understanding the pathogenesis of anthracnose and the immune mechanism of the hosts (Alkan et al., 2015; Zhang et al., 2018).

## Conclusions and future perspective

*Colletotrichum* spp.is one of the important pathogenic fungi with many species, hosts, and wide distribution. In recent years, the research on the control and pathogenic mechanism of *Colletotrichum* spp. has made good progress. In the future, the research on the control and pathogenic molecular mechanism of *Colletotrichum* spp. is still the focus. However, there is still a lack of safe and effective drugs or biological reagents to control oil tea anthracnose. Although the understanding of *Colletotrichum* spp. has made significant progress, the current research on molecular mechanism of anthracnose is obviously insufficient. There are only a few species mentioned above, such as *Co. gloeosporoides, Co. higginsianum*, and *Co. fruticola*. However, there are more other pathogenic molecular mechanisms of *Colletotrichum* spp. that have not been explored. Secondly, the research on the pathogenic molecular mechanism of anthracnose should gradually develop from the functional research of a single gene to the analysis of signal network, regulation mechanism, and omics. More importantly, there are few reports on the interaction mechanism between *Colletotrichum* spp. and hosts, especially the mechanism of oil tea responding to *Colletotrichum* spp. Exploring the pathogenic mechanism of anthracnose in oil tea is expected to provide reference for the green prevention and control of anthracnose in oil tea, and also helps people better understand the molecular mechanism of plant pathogen growth, development, and pathogenesis. At present, the prevention and control technology of oil tea anthracnose is still based on traditional agricultural control and chemical control. With the continuous innovation of technology and the demand for safe and efficient control technology, the development of biological control and seed selection of disease resistant varieties will be rapidly promoted.

## Author contributions

XinggC, GZ, and JL contributed to the conception of the manuscript. XinggC, XingzC, XM, and QT contributed significantly to manuscript preparation. All the authors have read and agreed to the published version of the manuscript.

## Funding

This research was funded by the National Natural Science Foundation of China (Grant No. 31971661); Postgraduate Scientific Research Innovation Project of Hunan Province (Grant No. CX20200712); Scientific Innovation Fund for Post-graduates of Central South University of Forestry and Technology (Grant No. CX20201008).

## Conflict of interest

The authors declare that the research was conducted in the absence of any commercial or financial relationships that could be construed as a potential conflict of interest.

## Publisher's note

All claims expressed in this article are solely those of the authors and do not necessarily represent those of their affiliated organizations, or those of the publisher, the editors and the reviewers. Any product that may be evaluated in this article, or claim that may be made by its manufacturer, is not guaranteed or endorsed by the publisher.
